# Human taste detection of glucose oligomers with low degree of polymerization

**DOI:** 10.1371/journal.pone.0183008

**Published:** 2017-08-29

**Authors:** Alexa J. Pullicin, Michael H. Penner, Juyun Lim

**Affiliations:** Department of Food Science & Technology, Oregon State University, Corvallis, Oregon, United States of America; Barnard College, UNITED STATES

## Abstract

Studies have reported that some animals, including humans, can taste mixtures of glucose oligomers (i.e., maltooligosaccharides, MOS) and that their detection is independent of the known T1R2/T1R3 sweet taste receptor. In an effort to understand potential mechanisms underlying the taste perception of glucose oligomers in humans, this study was designed to investigate: 1) the variability of taste sensitivity to MOS with low degree-of-polymerization (DP), and 2) the potential role of hT1R2/T1R3 in the MOS taste detection. To address these objectives, a series of food grade, narrow-DP-range MOS were first prepared (DP 3, 3–4, 5–6, and 6–7) by fractionating disperse saccharide mixtures. Subjects were then asked to discriminate these MOS stimuli as well as glucose (DP 1) and maltose (DP 2) from blanks after the stimuli were swabbed on the tongue. All stimuli were presented at 75 mM with and without a sweet taste inhibitor (lactisole). An α-glucosidase inhibitor (acarbose) was added to all test stimuli to prevent oral digestion of glucose oligomers. Results showed that all six stimuli were detected with similar discriminability in normal tasting conditions. When the sweet receptor was inhibited, DP 1, 2, and 3 were not discriminated from blanks. In contrast, three higher-DP paired MOS stimuli (DP 3–4, 5–6, and 6–7) were discriminated from blanks at a similar degree. Overall, these results support the presence of a sweet-independent taste perception mechanism that is stimulated by MOS greater than three units.

## Introduction

There is mounting evidence that humans [1,2] and other animals [3,4] are capable of tasting glucose oligomers and polymers, namely, maltooligosaccharides (MOS) and maltopoly-saccharides (MPS), and that this detection is largely independent of the known sweet receptor, T1R2/T1R3 [2,5–7]. Nearly all of these prior studies, however, have used MOS/MPS stimuli with disperse saccharide profiles, and/or appreciable amounts of simple sugars (e.g., Polycose; see [1] for detailed composition). Consequently, the use of such disperse stimuli has prohibited further interpretation as to which saccharides in stimuli mixtures were consciously tasted, or the mechanisms by which the perceptible stimuli elicited the taste response.

Two studies so far have made an effort to understand the target MOS/MPS that are being perceived by using stimuli with considerably less disperse degree-of-polymerization (DP) profiles. Note that MOS and MPS are defined herein to have a DP of 3–10 and DP > 10, respectively. Behavioral work by Sclafani and colleagues [8] reported that rats preferred a MOS stimulus composed of roughly equal amounts DP 4 to 8 over maltotriose (DP 3), maltose (DP 2), and glucose (DP 1). When comparing this MOS stimulus to a MPS mixture (average DP 43), rats significantly preferred the MOS over the MPS. Lapis et al. [2] reported similar trends when exploring taste perception of two mixed MOS/low-DP MPS stimuli (average DP 7 and 14) and a MPS stimulus (average DP 44) in humans. Specifically, subjects were able to detect the stimuli with the average DP 7 and 14, but not the average DP 44 stimulus at equimolar concentration, while oral digestion by salivary α-amylase was inhibited. These works together suggest that MOS and potentially low-DP MPS are the most probable stimuli eliciting taste perception.

Based on a number of genetic [5,7], behavioral [9,10], and human psychophysical studies [1,2], it is clear that the known sweet receptor, T1R2/T1R3, cannot fully explain the taste perception of MOS/MPS mixtures (see [2,11]). Interestingly, a recent study [6] suggested that maltotriose (DP 3) is detected through T1R2/T1R3 in mice. This result is intriguing because it has generally been assumed that mono- and disaccharides (i.e., simple sugars) are the only class of carbohydrate detected by this receptor. Pilot studies conducted in our laboratory suggested the same trend occurred in humans; this led us to raise the question of whether additional MOS, especially lower DP MOS, would also be detected, at least to some degree, by T1R2/T1R3.

In order to explore the possibility of a T1R2/T1R3-independent mechanism underlying MOS taste perception, at least three issues should be addressed. First, it is critical to further narrow down the DP ranges of target stimuli that are being tasted. Second, it is of interest to determine whether the specific DP ranges of target stimuli are detected to similar degrees and how these degrees of detection compare to that of simple sugars. Third, it is important to assess the potential role of T1R2/T1R3 in the detection of any target stimuli tested, especially low-DP MOS. These three issues are addressed in this paper. Due to a lack of commercially available MOS with specific, narrow DP profiles suitable for human testing, a series of food-grade MOS, having DP ranges of 3 to 7, were prepared and characterized herein. The results confirm that humans can taste pairs of glucose oligomers having a DP of 3 to 7, and that their stimulatory impact is similar to that of simple sugars on an equimolar basis. Furthermore, the results suggest that glucose oligomers greater than 3 units stimulate a taste perception pathway independent of T1R2/T1R3.

## Materials and methods

### Sample preparation and characterization

#### Materials

Maltodextrin (MD) was provided by Tate & Lyle Ingredients Americas, Decatur, IL (Corn syrup solids: STARDRI DE20). Solvents included ACS/USP-grade 100% ethanol (Pharmco Aaper, Shelbyville, KT) and deionized (DI) water (18.2Ω) produced using a Millipore Direct-Q 5 UV-R water purification system. Microcrystalline cellulose (Avicel PH 101) was purchased from FMC Corp. (Philadelphia, PA) and potato starch (Bob’s Red Mill, Milwaukie, OR) was purchased from a supermarket.

Thin layer chromatography (TLC) silica gel 60 plates were purchased from EMD Millipore (Billerica, MA). Carbohydrate standards for high performance liquid chromatography (HPLC) were purchased from the following vendors: glucose and maltose from Sigma Aldrich Corporation (St. Louis, MO); maltotriose and maltotetraose from Carbosynth Limited, (UK); maltopentaose, maltohexaose, and maltoheptaose from TCI America (Portland, OR). 1-napthol (ReagentPlus ≥99%) and butyl alcohol (≥99%, FCC, FG) were from Sigma-Aldrich (St. Louis, MO); sulfuric acid (ACS-grade anthrone, 99%) from Alfa Aesar (Ward Hill, MA); bicinchoninic acid sodium salt (BCA) from Pierce Chemical Co. (Rockford, IL); and deuterium oxide (99.96%) from Cambridge Isotope Laboratories (Tewksbury, MA).

#### Column-ready sample preparation

Two grams MD was dissolved in 3 mL DI water at 50–55°C with stirring. After complete dissolution, 9.5 mL ethanol was added in 1 mL increments with 1–2 min stirring/heating in between additions, allowing for increased precipitation of high-DP MOS/MPS. This resulted in a 76% ethanol/water mixture (all solvents described herein are on a v/v basis). The top liquid phase, containing the target low-DP oligomers (i.e., column-ready sample) was then pipetted off for loading onto the column. This step took advantage of the higher solubility of low-DP MOS (vs. the lower solubility of higher-DP MOS and MPS) in high ethanol solutions [12], thus allowing a majority of higher-DP MOS and MPS to precipitate out of solution, giving a sample enriched in low-DP components (i.e., DP 1–7).

#### MOS fractionation using starch/cellulose columns

A combination of cellulose and starch were chosen as stationary phase materials due to their low cost, food-safe nature, and MOS separation capabilities; specifically, potato starch was selected after comparing other starches (e.g., corn, tapioca) due to its larger granule size, which led to a better flow rate and thus minimized column running time. Pilot studies with the combined starch/cellulose stationary phases found proportions of 150g cellulose to 50g potato starch gave optimal results. To this cellulose/starch mixture, 75% ethanol was added and mixed to form a slurry, which was then funneled down the walls of a previously wetted column (dimensions: 73 mm I.D. x 305 mm length). The packed column was then rinsed with additional 75% ethanol, aided by compressed air (~1 psi), until the eluate went from deep yellow to colorless and a final column height of roughly 12 cm was reached. Column-ready sample preparations (~12 mL) were layered on the top of packed/washed columns, and step gradients, in percent ethanol, were then run as follows: 1.2 L of 90%, 1.2 L of 85%, 2.0 L of 80%, 1.0 L of 75%, and 0.5 L of 70%. Fractions of 100–300 mL (determined experimentally) were collected and their content was analyzed by spotting each fraction on thin layer chromatography (TLC) plates. These plates were developed in an enclosed chamber using an ethanol/water/butanol solvent system (69/17/14, respectively). Saccharides on developed plates were visualized using a staining solution described by Robyt & Mukerjea [13]. This step allowed for qualitative and rough quantitative estimates of the MOS character of each collected fraction.

#### Solvent removal and lyophilization

Removal of ethanol from the MOS preparations was critical to avoid any interference of residual ethanol with the sensory properties of the final MOS products. Ethanol removal was achieved by solvent removal followed by water washing three times using a rotary evaporator (Büchi Rotovapor R-205, Büchi Labortechnik AG) equipped with a 55° water bath (Buchi B-490) and vacuum pump (Chemglass Scientific Apparatus/10 Torr). Concentrated MOS preparations were stored at -23°C until drying by lyophilization in a VirTis CONSOL 4.5 freeze dryer.

#### High performance liquid chromatography (HPLC)

Lyophilized MOS samples were re-solvated and loaded onto a Prominence UFLC-HPLC system (Shimadzu, Columbia, MD) equipped with a system controller (CMB-20A), degasser (DGU-20A), solvent delivery module (LC-20AD), autosampler (SIL-10A), column oven (CT20-A), and evaporative light scattering detector (ELSD-LT II; kept at 60°C with nitrogen gas pressure of 350 kPa) on a combined Ag^2+^ polystyrene ion-exchange guard and analytical columns run at 80°C (Supelcogel, Hercules, CA). DI water was used as the mobile phase at a rate of 0.20 mL per minute. Standard curves were prepared using commercially available glucose/oligomer (DP 1–7) standards. LOD values for DP1-7 standards were found to be ≤ 0.006 mg/mL [12]. LCsolution computer software (Shimadzu, Kyoto, Japan) was used for peak integration.

#### Nuclear magnetic resonance (NMR)

NMR analyses were carried out 1) to verify the absence of ethanol from the preparations and 2) to determine the presence of α-1,6 linkages in the samples, characterized by a signal at 4.881–4.924 ppm [14]. A Bruker AVIII 700 MHz 2-channel spectrometer with a 5 mm dual carbon (DCH) cryoprobe with z-axis gradient was used to analyze samples at room temperature dissolved in D_2_O. Topspin 2.1 computer software was used to acquire spectra.

#### Reducing sugar assay

Reducing ends were quantified using the BCA/copper-based assay in order to determine average DP values of the MOS preparations. The protocol described in Balto et al. [12] was followed. A calibration curve was produced using aqueous maltose samples of 0 to 75 μM. Assays were done in triplicate.

#### Total carbohydrate assay

Total carbohydrate content was determined using the spectrophotometric anthrone/sulfuric acid assay described by Brooks and Griffin [15]. The protocol described in Balto et al. [12] was followed. A calibration curve was produced using aqueous glucose samples at 0 to 2 mg/mL. These values were multiplied by 0.90 to adjust for water of hydrolysis. Assays were done in triplicate.

### Psychophysical study

#### Subjects

Twenty-three subjects were recruited from Oregon State University and surrounding areas to participate in an initial screening session (see below). Of these, a total of 16 subjects (10 female, 6 male) between the ages of 21 and 59 (median = 27 yrs) qualified and participated in the following testing session. Participating subjects confirmed that they were non-smokers, not pregnant, not taking prescription pain medication or insulin; had no history of taste or smell loss or other oral disorders; had no oral lesions, canker sores, or piercings; and did not have a history of food allergies. Subjects were further asked to comply with the following restrictions prior to their testing session: 1) no dental work within 48 hours; 2) no alcohol consumption within 12 hours; 3) no consumption of acidic and/or caffeinated foods and beverages or dairy products within 4 hours; 4) no consumption of food or beverage except water within 1 hour; 5) no use of any menthol-containing products within 1 hour; and 6) no physically demanding activity within 1 hour. Subjects gave written consent and were paid for the sessions attended. The experimental protocol was approved by the Oregon State University Institutional Review Board and complies with the Declaration of Helsinki for Medical Research. The experimental protocol was also registered under the Clinical Trial registry (NCT02589353), www.clinicaltrials.gov.

#### Stimuli

In addition to the four MOS samples produced (DP 3, 3–4, 5–6, and 6–7), glucose (DP 1) and maltose (DP 2) were purchased separately from Spectrum Chemical Mfg. Corp. (New Brunswick, NJ). All six test stimuli were prepared as 75 mM aqueous solutions with corresponding water blanks; equimolar MOS stimuli were prepared from lyophilized MOS samples based on values calculated from the reducing sugar assay (Table 1). Based on our previous [2] and pilot studies, sugars and MOS samples at 75 mM elicit a weak but recognizable taste sensation for most (but not all) subjects. In addition, two stimuli were used for subject screening: 1) 75 mM DP 2 and 2) 150 mM glucose oligomer mixture (S1; prepared in Balto et al. [12]) with an average DP of 7 (composed of 75% DP 3–8, 25% DP 9+). All stimuli and water blanks included 5mM acarbose, which was found to be effective at inhibiting α-amylase [2]. Test stimuli and corresponding blanks were also prepared without and with 1.4 mM lactisole, which binds to the transmembrane region of hT1R3, effectively inhibiting sweet taste [16]. Although lactisole itself is tasteless, it has been known to elicit a sweet “water-taste” sensation when rinsed away from the receptor [17]; data suggest this “water-taste” effect is attenuated in colder temperatures [18]. Thus, all target stimuli, blanks, as well as rinse water were presented at 10°C to prevent sweet “water-taste”. Stimuli were prepared at least 16 h prior to the testing session to allow for complete mutarotation of glucose tautomers [19]. The stimuli were presented by swabbing technique (see below) to prevent any textural cues. Similarly, potential olfactory cues from the stimuli were controlled by using nose clips.

**Table 1:** Chemical characterization of four MOS samples prepared for psychophysical testing.

Sample	Percent Carbohydrate ^a^^,^^b^	moles RE^c^ per 100 gram ^a^^,^ ^d^	Linkage prevalence ^e^ (1,4-):(1,6-)
DP 3	99.7 ± 0.1	0.199 ± 0.004	ND ^f^
DP 3–4	98.2 ± 0.1	0.166 ± 0.004	ND
DP 5–6	99.8 ± 0.1	0.100 ± 0.010	ND
DP 6–7	99.3 ± 0.1	0.093 ± 0.002	ND

^a^ Values are means ± SD in triplicate, expressed on a dry weight basis.

^b^ Determined using anthrone/H_2_SO_4_-assay with glucose as a standard.

^c^ moles RE = moles of reducing ends

^d^ Determined using Cu/bicinchoninic acid-assay with maltose as a standard.

^e^ Determined from NMR spectra.

^f^ ND = α-1,6 linkage signal not detected in NMR spectrum.

#### Psychophysical test

An initial screening session was included to confirm that all subjects could detect a simple sugar as well as glucose oligomers at a relatively low concentration (i.e., 75 mM). During this session, two target stimuli (75 mM DP 2 and 150 mM S1) were given to subjects four times each, in counterbalanced blocks, in the form of a triangle test. Thus, subjects were given a total of eight trials. Prior to testing, subjects were verbally instructed on the task they were to perform, rinsed their mouths three times with room temperature water, and put on a nose clip. Subjects were then instructed to extend their tongue out of the mouth and hold it steady between the lips. Three stimuli (one target and two water blanks) were presented, one at a time, to the subject by swabbing across the tip of the tongue three times (volume of stimuli absorbed by the swab was approximately 0.15 mL) with rinsing in between. After all three stimuli in the set had been received, subjects were asked to verbally identify which stimulus of the three was different. A one minute break was given between trials, during which subjects were instructed to rinse their mouth three times with water. In order to qualify for subsequent low DP MOS discrimination testing, subjects were required to correctly discriminate the target stimulus from the blanks three out of four times (for both DP 2 and S1). In cases where the subject was nearing qualification but did not meet the above stated criteria, an additional trial for the incorrectly identified stimulus was provided. In total, 16 of the 23 subjects qualified for the testing session.

For the testing session, six stimuli were presented in two blocks (without and with lactisole), giving a total of 12 trials; the presentation order of the blocks, the order of the target stimuli within the blocks, and the three stimuli within each triangle test, was randomized across subjects. Triangle tests were carried out as described for the screening session. During the test session, subjects were asked to provide their responses on a paper ballot. In addition to the one minute break between trials, a three minute break was given between blocks, during which subjects were asked to rinse their mouth three times with water.

#### Data analysis

The number of correct identifications among all subjects for each target stimulus was tallied and converted to a proportion correct. The proportions were then used to find corresponding *d’* values by consulting the *d’* table for a triangle test [20]. The *d’* value represents the degree of perceptible difference between two stimuli, and specifically, is a measure of the separation of the signal (stimulus) and noise (blanks) distributions in terms of the standard deviations of the distributions [21]. The *d’* analysis was then used to determine if the target stimuli were significantly discriminated at p < 0.05.

## Results

### Sample MOS characterization

**Fig 1:**
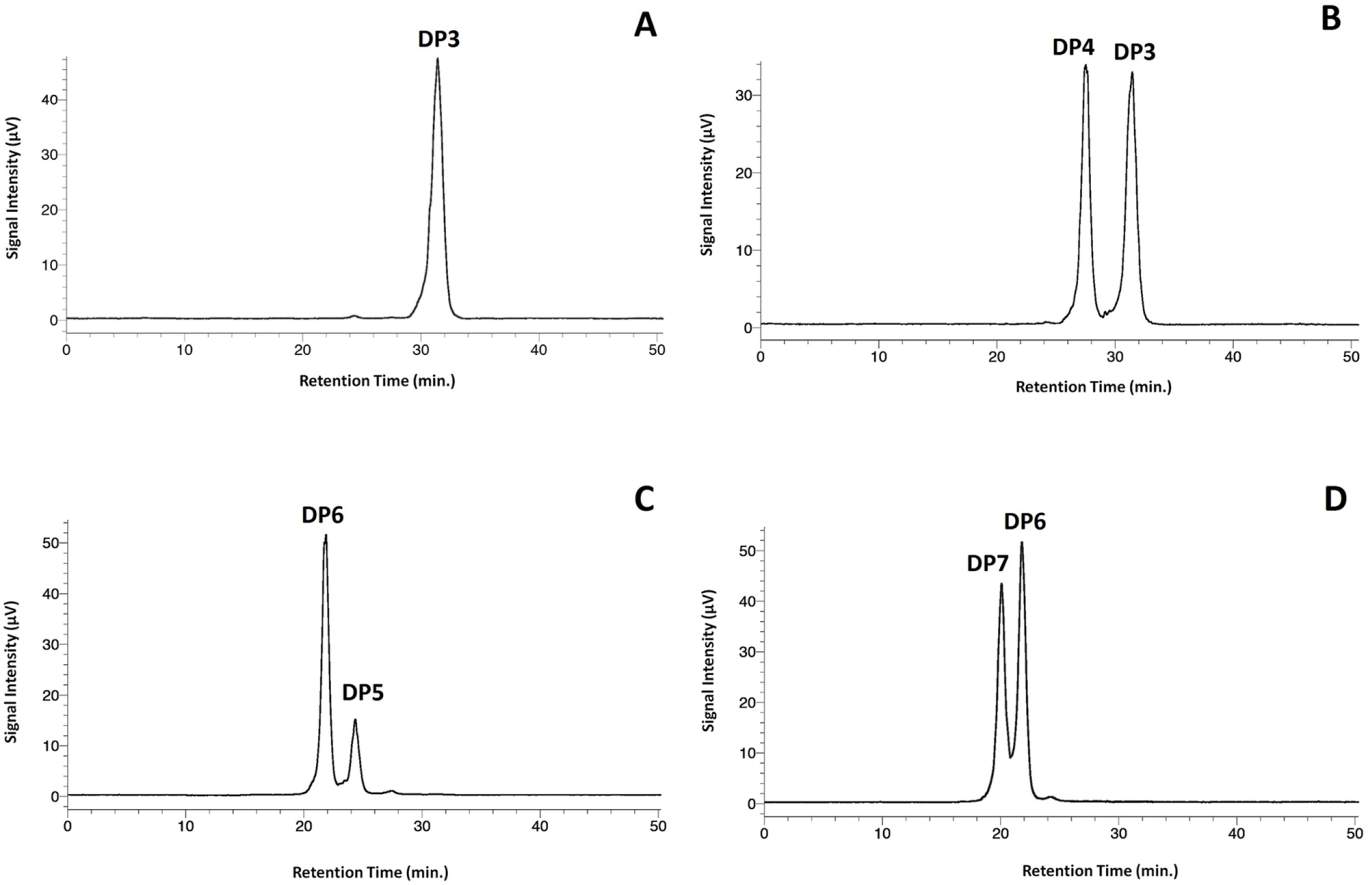
Representative chromatograms from HPLC-ELSD depicting saccharide character of the four samples (A, DP 3; B, DP 3–4, C: DP 5–6, and D: DP 6–7) produced through column chromatography.

MOS preparation described above led to one isolate and three glucose oligomer pairs. Chemical characteristics are described in Table 1. The carbohydrate content of all four samples was found to be > 98% (Table 1). The DP 3 sample consisted of only maltotriose, supported by both the reducing sugar analyses (Table 1) and HPLC data (Fig 1A). Whereas the DP 3–4 and DP 6–7 samples were composed of roughly equivalent quantities of neighboring chain-lengths, the DP 5–6 sample was notably heavier in DP 6 versus DP 5. The saccharide compositions of the three paired MOS samples can be easily visualized in Fig 1B, 1C and 1D. NMR analyses indicate the removal of ethanol from the preparations and the linear nature of the saccharides making up the four samples (i.e., only α-1,4-linkages were detectable; see S1 Fig in Supplemental materials for spectra).

### Psychophysical results

Table 2 shows the proportion of subjects to correctly identify the target stimulus from blanks, along with corresponding detectability (*d’* values) of the six test stimuli in the absence and presence of lactisole. Results indicate that all target stimuli were significantly discriminated from blanks in the absence of lactisole, i.e., under the normal tasting condition. Moreover, *d’* values between all six stimuli were found to be statistically insignificant from each other (p > 0.05, the number of correct responses ranged from 11 to 13 out of 16), suggesting all stimuli were detected at a similar degree. In the presence of lactisole, subjects were not able to discriminate DP 1, DP 2, or DP 3 from blanks (p > 0.05). In respect to the MOS of DP 4–7 (i.e., DP 3–4, DP 5–6, and DP 6–7), however, all remained significantly discriminable even in the presence of lactisole. The three higher-DP MOS-containing samples were detected similarly within lactisole treatments (p > 0.05).

**Table 2:** Proportion correct^a^ and discriminability (*d’* value)^b^ of target stimuli in the absence and presence of lactisole.

*d'*	DP 1	DP 2	DP 3	DP 3–4	DP 5–6	DP 6–7
Lactisole absent	^a^ 0.69 ^b^ 2.45**	0.69 2.45**	0.69 2.45**	0.81 3.20**	0.75 2.78**	0.75 2.78**
Lactisole present	0.25 0	0.25 0	0.31 0	0.63 2.13*	0.63 2.13*	0.63 2.13*

Discrimination results (proportion correct and calculated *d’* value) from triangle tests performed using six stimuli at 75 mM. All stimuli were prepared with 5 mM acarbose, without or with 1.4 mM lactisole.

DP = degree of polymerization.

*,** p-values significant at 0.001 and 0.0005, respectively by *d’* analysis.

## Discussion

### Characterization of food-grade MOS samples

In order to achieve the primary goal of this study, it was necessary to produce a series of food-grade MOS with narrow, well-defined saccharide profiles. By applying the described food-safe fractionation protocol to MD preparations, we obtained isolated DP 3 and neighboring pairs of low-DP MOS (i.e., DP 3–4, 5–6, and 6–7). These MOS samples were found to be of high purity (>98%) and contained no detectable amounts of ethanol remaining from the fractionation steps; thus, sensory cues that could be imparted by ethanol or other impurities in the stimuli were negligible. NMR spectra indicated all sample preparations were composed of isomerically pure (linear) MOS. This property is optimal for studies investigating receptor-ligand interactions, where stimuli containing mixed linear and branched MOS would complicate interpretations.

### Humans can taste glucose oligomers of DP 3–7

The findings of the current study, which employs the narrowest MOS stimuli tested in humans to date, provide further psychophysical evidence that humans can taste glucose oligomers of DP 3 to 7. These findings are consistent with the previous report that tested less refined MOS preparations in humans [2]. Note that any potential olfactory and textural cues were carefully controlled, and hence these sensory cues were not responsible for the stimuli detection. In normal tasting circumstances, introduction of glucose oligomers and polymers into the oral cavity results in hydrolysis of the α-1,4 linkages between glucose residues, principally through the action of α-amylase in saliva [22,23]. To keep the saccharide profiles of the MOS preparations intact, all stimuli were prepared with acarbose, an α-amylase inhibitor. We were thus able to infer that taste detection was due to the target stimuli proposed.

When the degrees of detectability between glucose oligomers and simple sugars were compared, the *d’* values were statistically insignificant from one another (see Table 2, lactisole absent condition). This finding is consistent with Lapis et al. [2], where a MOS preparation (average DP 7) gave a nearly equivalent dose-response function to that of glucose when the two stimuli were compared on an equimolar basis from 45 mM to 225 mM. *d’* values between the three paired MOS stimuli (DP 3–4, 5–6, and 6–7) were also found to be statistically insignificant (Table 2), suggesting that each of the MOS stimuli tested had a similar stimulatory impact on the mode of perception when considered on an equimolar basis (75 mM). It remains unknown as to whether isomers of linear MOS DP 3–7 (i.e., non-linear saccharides containing α-1,6 branch points) would give similar results; the taste perception of branched saccharides was not addressed herein. Furthermore, it is unknown whether taste sensitivity to MOS differs between the anterior and posterior parts of the tongue. It has been shown in rats that transection of the chorda tympani nerve, which innervates the anterior tongue, suppressed sucrose and polycose intakes to a similar degree, whereas transection of the glossopharyngeal nerve, which innervates the posterior tongue, selectively reduced polycose intake [24]. The possible differential sensitivity of MOS based on locus of stimulation in humans is currently being investigated.

### Taste detection of maltotriose is through hT1R2/T1R3

In order to investigate the potential role that hT1R2/T1R3 receptors play in MOS taste detection, lactisole was used as a means to block normal hT1R2/T1R3 function. Accordingly, subjects were unable to detect mono- and disaccharides (glucose and maltose, respectively) in the presence of lactisole (i.e., the detection rate was below the chance level, 33% for a triangle test). In contrast, the subjects still could detect the paired glucose oligomer stimuli (i.e., DP 3–4, 5–6, and 6–7) in the presence of lactisole; in fact, the detectability of the paired glucose oligomer stimuli between the two lactisole conditions were statistically indifferent (*p* > 0.05), although the presence of lactisole somewhat lowered their detection rates (see Table 2; 75~81% to 63%). This slight reduction in detectability of the paired stimuli has at least two potential explanations: 1) hT1R2/T1R3 may contribute to (but neither be necessary nor sufficient for) the detection of MOS > DP 4, or 2) lactisole may interfere with the MOS detection mechanism.

Most importantly, significant discrimination of DP 3 (maltotriose) was no longer observed when hT1R2/T1R3 was blocked. Consequently, we conclude that the taste detection of maltotriose, but not the higher-DP glucose oligomers tested, is mediated by hT1R2/T1R3. This result supports studies using T1R2/T1R3 KO mice, where responses to maltotriose were severely impaired in the absence of either component of the receptor [6]. This observation is worth noticing, given that maltose and maltotriose are the final products of α-amylase hydrolysis [25].

Another interesting observation is that the discriminability of DP 3–4 remained similar to that of the other, higher DP MOS tested despite taste discrimination of DP 3 alone was not significant in the presence of lactisole. This outcome is surprising, given that the DP 3–4 stimulus consists of roughly 50% DP 3. There are two possibilities to explain this observation. First, it is possible that the stimulating effect of DP 4 is greater than that of the other MOS tested. Second, the concentration used in this study was sufficiently high that relatively small differences in discriminability between the higher DP MOS were not achievable.

### Potential taste mechanisms of glucose oligomers greater than DP 3

Two mechanisms separate from the T1R2/T1R3 sweet receptor have been suggested to explain taste detection of glucose oligomers. The first suggestion is based on glucose oligomer catabolism. Some glucosensors, namely, glucose transporters (GLUTs), the sodium-glucose cotransporter 1 (SGLT1), and the ATP-gated K^+^ (K_ATP_) channel are expressed in T1R3 taste cells [26,27] of mice. Importantly, α-glucosidases expressed in taste cells (e.g. maltase-glucoamylase) along with salivary α-amylase serve to break down the α-glucosidic bonds of glucose oligomers/polymers to liberate glucose for these pathways when carbohydrate-containing foods are consumed. Based on these findings, it is reasonable to consider that this mechanism may be involved in the taste perception of glucose oligomers [25]. If this were the case, we would expect to see similar responses to glucose as we would with glucose oligomers when they are discriminated against water when T1R2/T1R3 is blocked. This hypothesis, however, does not appear to be fully supported by human psychophysical studies conducted here or in previous work [2]; rather, the ability to discriminate glucose-containing solutions from water is greatly diminished in the absence of normal T1R2/T1R3 function. Supporting this idea, Glendinning and colleagues reported that K_ATP_ channels are necessary for taste-mediated cephalic phase insulin release, but not for taste-mediated behavioral attraction of mice to sugars [28,29].

A second possibility to explain the conscious perception of glucose oligomers is the presence of a novel taste transduction mechanism, originally hypothesized by Nissenbaum and Sclafani [10] to exist in rodents. When consumed, starch is rapidly hydrolyzed to glucose oligomers/polymers of various DP and maltose [30, 31]. A taste perception mechanism for MOS seems plausible, given the importance of starch in mammalian diets. A mechanism that evolved to detect these types of hydrolysis products would presumably be highly beneficial to rapidly identify food sources rich in digestible carbohydrates in nature.

Taste quality data were not collected for the individual MOS stimuli tested in this study. However, in our previous study [2], subjects described mixtures of MOS as “starchy,” that is different from the sweet taste of natural and artificial sugars and sweeteners. The extent to which and the mechanism by which MOS perception contributes to the overall flavor of foods and beverages remain unanswered questions. Overall, the current results support the proposed novel taste perception mechanism in humans stimulated by glucose oligomers greater than three units. The sensory mechanisms underlying all gustatory detection, including the perception of sugars and starch hydrolysis products, are not fully understood. Current data support the premise that multiple gustatory pathways are involved in carbohydrates sensing.

## Supporting information

S1 Fig Representative ^1^H NMR spectra of MOS preparations (A: DP 3; B: DP 3–4; C: DP 5–6; D: DP 6–7) dissolved in D_2_O showing full and magnified spectra of the ppm range containing peaks corresponding to α-1,4 (5.305–5.395 ppm) and α-1,6 (4.881–4.924 ppm) linkages (circled). Absence of ethanol signal (1.1 ppm) circled.
(JPG)
