# Supplementary figures and images for: Human taste detection of glucose oligomers with low degree of polymerization
Source: PLoS One. 2017 Aug 29;12(8):e0183008. doi: 10.1371/journal.pone.0183008 (PMC5574539; doi:10.1371/journal.pone.0183008)

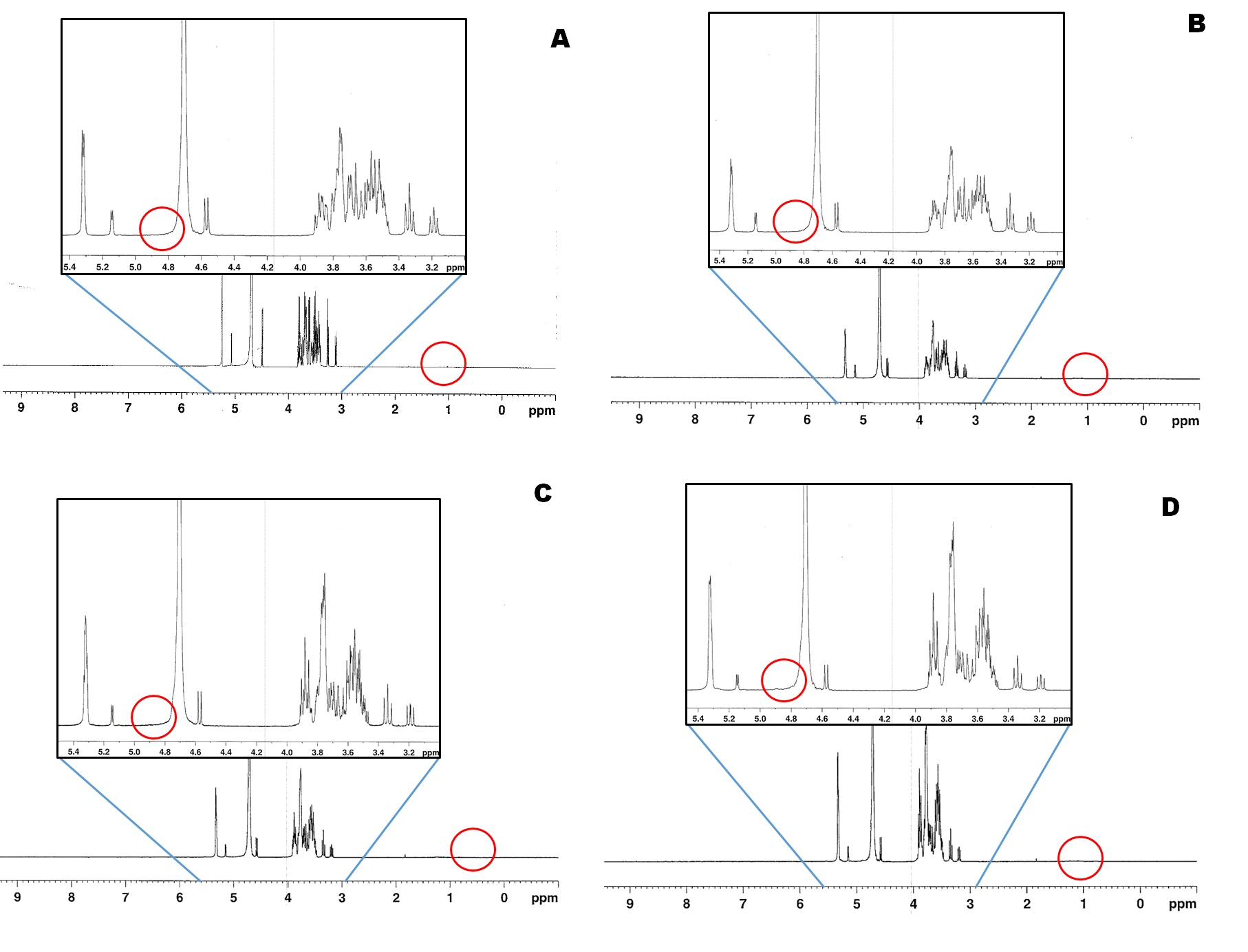

Supplement: S1 Fig — Absence of ethanol signal (1.1 ppm) circled. (JPG) [file pone.0183008.s001.jpg]
